# Unraveling the evolutionary dynamics of the *TPS* gene family in land plants

**DOI:** 10.3389/fpls.2023.1273648

**Published:** 2023-10-12

**Authors:** Xue-Mei Yan, Shan-Shan Zhou, Hui Liu, Shi-Wei Zhao, Xue-Chan Tian, Tian-Le Shi, Yu-Tao Bao, Zhi-Chao Li, Kai-Hua Jia, Shuai Nie, Jing-Fang Guo, Lei Kong, Ilga M. Porth, Jian-Feng Mao

**Affiliations:** ^1^ National Engineering Research Center of Tree Breeding and Ecological Restoration, State Key Laboratory of Tree Genetics and Breeding, Key Laboratory of Genetics and Breeding in Forest Trees and Ornamental Plants, Ministry of Education, College of Biological Sciences and Technology, Beijing Forestry University, Beijing, China; ^2^ Shuangyushu No.1 Primary School, Beijing, China; ^3^ Key Laboratory of Crop Genetic Improvement & Ecology and Physiology, Institute of Crop Germplasm Resources, Shandong Academy of Agricultural Sciences, Jinan, China; ^4^ Rice Research Institute, Guangdong Academy of Agricultural Sciences & Key Laboratory of Genetics and Breeding of High Quality Rice in Southern China (Co-construction by Ministry and Province), Ministry of Agriculture and Rural Affairs & Guangdong Key Laboratory of New Technology in Rice Breeding, Guangzhou, China; ^5^ Department of Horticulture and Food, Guangdong Eco-Engineering Polytechnic, Guangzhou, China; ^6^ Personnel Section, Qufu Nishan National Forest Park Management Service Center, Qufu, China; ^7^ Départment des Sciences du Bois et de la Forêt, Faculté de Foresterie, de Géographie et Géomatique, Université Laval Québec, Québec, QC, Canada; ^8^ Department of Plant Physiology, Umeå Plant Science Centre, Umeå University, Umeå, Sweden

**Keywords:** terpene synthases, horizontal gene transfer, gene fusion, synteny network, biosynthetic gene clusters

## Abstract

Terpenes and terpenoids are key natural compounds for plant defense, development, and composition of plant oil. The synthesis and accumulation of a myriad of volatile terpenoid compounds in these plants may dramatically alter the quality and flavor of the oils, which provide great commercial utilization value for oil-producing plants. Terpene synthases (*TPSs*) are important enzymes responsible for terpenic diversity. Investigating the differentiation of the *TPS* gene family could provide valuable theoretical support for the genetic improvement of oil-producing plants. While the origin and function of *TPS* genes have been extensively studied, the exact origin of the initial gene fusion event - it occurred in plants or microbes - remains uncertain. Furthermore, a comprehensive exploration of the *TPS* gene differentiation is still pending. Here, phylogenetic analysis revealed that the fusion of the *TPS* gene likely occurred in the ancestor of land plants, following the acquisition of individual C- and N- terminal domains. Potential mutual transfer of *TPS* genes was observed among microbes and plants. Gene synteny analysis disclosed a differential divergence pattern between TPS-c and TPS-e/f subfamilies involved in primary metabolism and those (TPS-a/b/d/g/h subfamilies) crucial for secondary metabolites. Biosynthetic gene clusters (BGCs) analysis suggested a correlation between lineage divergence and potential natural selection in structuring terpene diversities. This study provides fresh perspectives on the origin and evolution of the *TPS* gene family.

## Introduction

Terpenes and terpenoids, constitute the largest and most structurally diverse group of natural compounds in plants and play crucial roles in various physiological and biochemical processes (Bohlmann et al., 1998; Yang et al., 2020; Yu et al., 2020; Zhou and Pichersky, 2020). These plant-derived terpenes offer a wide array of commercially and industrially viable renewable resources for the production of fragrances, flavors, essential oils, and medicinal properties (McGarvey and Croteau, 1995; Chaw et al., 2019). Plant volatile terpenoids (PVTs), largely consisting of monoterpenes and to a lesser extent the sesquiterpenes, are found in high concentrations in oil-producing plants (Little and Croteau, 1999; Li et al., 2021). This great concentration and diversity of PVTs gives plant oils varied properties and flavors, which has led to the extensive utilization of oil-producing plants.

In fact, the PVTs constitute the major, and often the characteristic, components of essential oils (Little and Croteau, 1999; Li et al., 2021). *Eucalyptus* leaves are enriched in volatile terpenes such as 1-8 cineole, α-terpinene, γ-terpinene, carvacrol, thymol and limonene (King et al., 2006; Naidoo et al., 2014; Kainat et al., 2019). Citronellol, geraniol, and nerol are the three major components of rose oil (Li et al., 2021). The mint family, Lamiaceae, produces mint oils that are rich in monoterpenes and sesquiterpenes (Vining et al., 2017). Tea tree oil, another terpene-rich essential oil, is commonly used in cosmetics and skin care products due to its antimicrobial properties (Carson et al., 2006). Significant cases of terpene richness can also be found in conifer leaves and wood, which contain abundant monoterpenes and diterpenoids (Trapp and Croteau, 2001), as well as in yellowhorn (*Xanthoceras sorbifolium*), an underutilized oil-producing tree that yields triterpenoids (Wang et al., 2017; Chen et al., 2020).

The enormous diversity of plant terpenes in specialized metabolism can be primarily attributed to the diverse terpene skeletons generated by typical plant terpene synthases (*TPSs*) (Tholl, 2015; Alquézar et al., 2017; Pichersky and Raguso, 2018). Through studying the polymorphism of *TPS* genes, genetic improvement can be carried out to breed oil-producing plant varieties with high volatile terpenoid compound content. The *TPSs* belong to a mid-sized gene family and are prevalent in land plants (Chen et al., 2011; Jia et al., 2022). Each full-length plant *TPS* contains two conserved domains identified by their Pfam models: PF03936 (C-terminal) and PF01397 (N-terminal) (Chen et al., 2021). Based on the reaction mechanism and the products formed, *TPS* enzymes can be classified into two classes. Class I enzymes active sites, which are located in the C-terminal region, are characterized by highly conserved aspartate-rich DDxxD and “NSE/DTE” motifs found within an “*α*-domain”. In contrast, the active site of Class II enzymes is located in the N-terminal region between a pair of alphahelical double-barrel domains, known as the “*β-*domain” and an additional “*γ-*domain”, this site utilizes a “DxDD” motif (Yang et al., 2020; Zhou and Pichersky, 2020; Jia et al., 2022). In some non-seed land plants, *ent*-copalyl diphosphate/*ent*-kaurene synthase (*CPS/KS*) functions as a bifunctional “*αβγ*-tridomain” enzyme with both class I and class II activity at the N-terminal and C-terminal, respectively (Hayashi et al., 2006; Jia et al., 2018; Yang et al., 2020). This gene can synthesize both *CPS* and *KS*, which are the two key di-*TPSs* that sequentially catalyze the biosynthetic intermediate *ent*-kaurene of gibberellic acids (GAs) (Morrone et al., 2009; Kawaide et al., 2011). However, seed plants possess separate genes for *CPS* and *KS* synthesis but still contain the ancestral “*αβγ*-tridomain” architecture (Köksal et al., 2011; Zi et al., 2014; Jia et al., 2022). Consequently, the bifunctional “*αβγ*-tridomain” *TPS* is widely believed to be an ancestral gene.

Based on the structural and functional similarity between plant diterpene cyclases and bacterial diterpene cyclases, it is believed that the ancestral land plant “*αβγ-*tridomain” (*CPS/KC*) gene evolved through the fusion of the “*βγ*-didomain” (*CPS*) and the “*α*-domain” (*KS*). Subsequent loss of the “*γ-*domain” in the “*αβγ*-tridomain” led to the origin of the “*αβ*-didomain” genes (Cao et al., 2010; Köksal et al., 2011; Smanski et al., 2012). However, it remains unclear whether the fusion of the “*βγ*-didomain” (*CPS*) and the “*α*-domain” (*KS*) occurred in the ancestral land plants after acquiring them through horizontal gene transfer (HGT) from bacteria, or if the fusion first took place in microbes and was then acquired by ancestral land plants via HGT (Jia et al., 2022). Thus, phylogenetic analysis with comprehensive sampling of terpene synthase sequences from plants and microbes is necessary.

The early evolution of the plant *TPS* family has been reported with substantial evidence. It is likely that the ancestral bifunctional *TPS* gene underwent at least two duplications, giving rise to three ancient *TPS* lineages that led to the present subfamilies TPS-c, TPS-e/f, and TPS-h/d/a/b/g (Jia et al., 2022). The separate *CPS* genes (class II), along with the extant *CPS/KS* genes, form the TPS-c subfamily; the separate *KS* genes (class I) gave rise to the TPS-e/f subfamily. Meanwhile, the angiosperm-specific TPS-a/b/g, gymnosperm-specific TPS-d, and TPS-h subfamilies are dedicated to secondary metabolism and synthesize mono-, sesqui-, diterpenes, among others (Yang et al., 2020; Jia et al., 2022). The conservation and differentiation dynamics of *TPS* subfamilies involved in primary and secondary metabolism across plant lineages are still opaque. Examining the gene synteny of *TPS* genes across various species could provide crucial information to answer fundamental questions about the evolution of this important gene family (Zhao et al., 2017).

In eukaryotic organisms, nonhomologous genes that jointly encode the biosynthetic enzymes in a specialized metabolic pathway are often co-localized within the genome. These local clusters of genes are referred to as biosynthetic gene clusters (BGCs) (Kautsar et al., 2017; Nützmann et al., 2018; Rokas et al., 2018; Witjes et al., 2019). In plants, species-specific BGCs are formed through processes such as gene duplications, neofunctionalizations, subfunctionalizations, and relocations. These functional units are inherited and provide a selection advantage in response to various biotic stresses (Qi et al., 2004; Zhou et al., 2016; Wu et al., 2022). Some studies on terpene-related BGCs demonstrated that minor differences in the structures of the pathway end-products leading to diversification have arisen from gene duplication, random mutations, and neofunctionalization (Zhou et al., 2016; Nützmann et al., 2018; Rokas et al., 2018; Liu et al., 2020). Comparing a candidate BGC with homologous genomic loci across multiple plants can provide important information about its evolutionary conservation or diversification (Kautsar et al., 2017). Although BGC studies have recently received increasing attention and have been conducted in some plants, interspecific terpene-related BGC homology analysis is still lacking.

Here, we conducted a comprehensive investigation into the origin and evolutionary dynamics of plant *TPS* genes by utilizing a broad phylogenetic sampling and gene synteny-based comparative genomic analysis. Our results revealed potential mutual transfer of *TPS* genes between microbes and plants. Additionally, we found it likely that the PF03936 (C-terminal) and PF01397 (N-terminal) domains were individually acquired from microbes through HGT, and their fusion event probably occurred in the ancestor of land plants. Moreover, our comparative genomic analysis uncovered notable patterns, where the subfamilies involved in primary metabolism were conserved, while the subfamilies exclusively associated with secondary metabolite production exhibited significant divergence and radiation. We also observed a substantial expansion of *TPS* genes and the insertion or deletion of other metabolic genes in both homologous and species-specific BGCs. These findings enhance our understanding of plant terpenes diversity from multiple perspectives and provide insights relevant to studying the evolution of *TPS* gene families. Furthermore, our results offer valuable references for breeding oil-producing plants that contain high concentrations of volatile terpenoids.

## Materials and methods

### Species selection and gene identification

We analyzed assembled genome sequences and their annotated protein sequences from 74 plant species to identify *TPS* genes. The sampled plant species spanned diverse lineages, including Chlorophyta, Streptophyta, Bryophytes (Liverworts and Mosses), Lycopodiophyta, Gymnospermae (Cupressales, Gnetales, Ginkgoales), and Angiosperm (Apiales, Asterales, basal Angiosperms, Brassicales, Lamiales, Laurales, Malpighiales, Myrtales, Poales, Rosales, Sapindales, Solanales, Vitales) (**Supplementary Table 1**). We employed the hmmscan v3.2.1 (Mistry et al., 2013) with an E-value of 1e-5 to identify candidate *TPS* genes containing at least one conserved Pfam (Bateman et al., 2004) domain (PF01397 and/or PF03936). Subsequently, we verified the conserved domains via Conserved Domain Database (CDD; https://www.ncbi.nlm.nih.gov/Structure/cdd/wrpsb.cgi). Based on the presence of both PF01397 and PF03936 domains, only the PF01397 domain, or only the PF03936 domain, we classified these high-confidence *TPS* homologies into three categories: full-length *TPS* genes, PF01397-domain-genes, and PF03936-domain-genes.

To extensively mine *TPS* sequence among various microbes, we identified putative *TPS* sequences by searching the significantly matched proteins (containing at least one conserved domain PF01397 and/or PF03936) in NR databases using hmmscan v3.2.1 (Mistry et al., 2013) (E-value = 1e-5). The NR database were obtained from https://ftp.ncbi.nlm.nih.gov/blast/db/FASTA/ (accessed date: 2022-10-31). We separately extracted all protein sequences of bacteria, fungi and archaea from the database using TaxonKit, a command-line interface tool for handling NCBI taxonomy data (Shen and Ren, 2021). We extended the classification method of plant *TPS* genes to include sequences from bacteria, fungi and archaea. Sequences that matched either the PF01397 or PF03936 domains were classified into full-length *TPS* genes, PF01397-domain-sequences and PF03936-domain-sequences. Furthermore, we determined the species’ taxonomic origins of PF01397-domain-sequences and PF03936-domain-sequences using the taxonomizr package (versions 0.9.3, https://www.rdocumentation.org/packages/taxonomizr/versions/0.9.3) in R. The original taxon was listed in a matrix that included six hierarchical levels, i.e., phylum, class, order, family, genus, and species.

### Phylogenetic reconstruction

To classify the full-length *TPS* genes from plants, we separately constructed phylogenetic trees for full-length *TPS* genes from 62 angiosperm species and non-angiosperm species, including *T. wallichiana*, *Sequoiadendron giganteum*, *G. montanum*, *Ginkgo biloba*, *M. polymorpha*, *P. patens*, and *S. moellendorffii*. To investigate the origins of two full-length *TPS* genes (*WP_145718914.1* and *WP_260645699.1*) identified in two bacteria, we further performed phylogenetic reconstruction for these two genes in conjunction with 40 plant genes, and conservation motifs of these genes were identified using MEME (https://meme-suite.org/meme/tools/meme) (Bailey et al., 2009). Sequences alignment was prepared using MAFFT v7.407 (Katoh et al., 2002; Katoh and Standley, 2013) with “–anysymbol”, and poorly aligned regions were trimmed using trimAl v1.2.rev59 (Capella-Gutiérrez et al., 2009) with “-gt 0.4 -st 0.001”.

To explore the origin and relationships of the PF03936 and PF01397 domains in *TPS* genes across plants and microbes, we constructed separate unrooted phylogenetic trees using sequence data of these domains extracted from plants, fungi, archaea and bacteria. We clustered the sequences (size >100 aa) of PF03936 and PF01397 domains with at least 80% identity using CD-HIT with parameters -c 0.80 -n 5 -M 16000 -d 0 -T 8 and selected the longest sequences in each cluster for further analysis. For the PF03936 domain, we constructed a phylogenetic tree using representative sequences from archaea and plants, and randomly sampled 1000 sequences from bacteria and fungi using seqkit (Shen et al., 2016). For the PF01397 domain, we used all representative sequences from bacteria, archaea, fungi, and plants to construct the phylogenetic tree. Sequences alignment was prepared using MAFFT v7.407 (Katoh et al., 2002; Katoh and Standley, 2013) with “–anysymbol” option and without trimming.

We constructed all phylogenetic trees using either alignments or trimmed alignments, utilizing IQ-TREE v2.0.3 (Nguyen et al., 2014) with 1,000 replications of ultrafast bootstrap and Shimodaira-Hasegawa-like approximate likelihood-ratio (SH-aLRT) test. The best-fit models chosen by ModelFinder (Kalyaanamoorthy et al., 2017) were as follows: “JTT+R7” for the angiosperm full-length genes tree, “JTT+F+R6” for the non-angiosperm full-length genes tree, “JTT+F+R3” for the full-length bacterial genes tree, “LG+R10” for the PF03936 domain sequences tree, and “JTT+F+R6” for the PF01397 domain sequences tree. We interpreted and visualized those phylogenetic trees using the online tool iTOL v6 (Letunic and Bork, 2007).

### Two full-length *TPS* genes (*WP_145718914.1* and *WP_260645699.1*) from soil bacteria and further confirmation

Two full-length *TPS* genes (*WP_145718914.1* and *WP_260645699.1*) were identified from soil bacteria. To further confirm their occurrence, we conducted a homology analysis of their flanking regions among the conspecific and congeneric genomes. Homologous search was conducted by BLASTn, and significant match was identified for an E-value of less than 1e-5 and identity greater than 70%. The homologous analysis for *WP_145718914.1* (found in VLLG01000006.1 sequence of *C. japonensi*) was conducted among 41 *Chitinophaga* species, and the analysis for *WP_260645699.1* (found in JAOBSP010000025.1 sequence of *S. aureus*) was conducted among 96 *S. aureus* strains and 59 *Staphylococcus* species. The sequence homology of this flanking region analysis was visualized using the command ‘python -m jcvi.graphics.synteny’ of MCscan (Python version) (Wang et al., 2012).

### Synteny network analyses and phylogenetic profiling

We constructed a genomic synteny network of plant *TPS* genes based on the processes designed in the SynNet pipeline (Zhao et al., 2017; Zhao and Schranz, 2019). First, we performed a reciprocal all-against-all BLAST search using Diamond v0.9.22.123 (Buchfink et al., 2015) with -k 5 against sequences in the proteomes of 74 studied plants (**Supplementary Table 1**). After that, the genomic collinearity between all possible pairwise genome combinations using MCScanX (Tang et al., 2008; Wang et al., 2012) with default parameters (minimum match size for a collinear block = 5 genes, max gaps allowed = 25 genes) were calculated. Subsequently, we extracted all possible syntenic gene pairs of the full-length *TPS* genes, PF03936-domain-genes and PF01397-domain-genes to construct their respective synteny networks.

We further imported the full-length *TPS* gene synteny network into CFinder v.2.0.6 to detect potential *k*-clique communities (subnetworks) with *k*=3 (Derényi et al., 2005; Palla et al., 2005; Fortunato, 2010). The *k*-clique corresponds to the two-by-two connection of *k* nodes to each other (e.g., a *k*-clique of *k*=3 is equivalent to a triangle) (Zhao et al., 2017). After *k-*clique percolation, we visualized all networks using Gephi v0.9.2 (Bastian et al., 2009), considering *TPS* subfamily and plant lineages information for downstream analysis. We clustered the synteny networks of full-length *TPS* genes, PF03936-domain-genes and PF01397-domain-genes using infomap (Rosvall and Bergstrom, 2008), and counted the number of genes in resulting clusters. These derived gene numbers were mapped back to the 74 species (**Supplementary Table 1**). Angiosperms were arranged according to their phylogenetic treatment developed in APG (Angiosperm Phylogeny Group) IV (The Angiosperm Phylogeny et al., 2016), while the gymnosperms and early land plants arranged according to the previous studies (Cheng et al., 2019; Zhang et al., 2020).

### Identification of syntenic terpene-related BGCs among species

We identified BGCs from the whole genomes of our 74 sampled plants (**Supplementary Table 1**) using antiSMASH v3.0.5 (PlantiSMASH python version) (Kautsar et al., 2017) with the parameters of “–taxon plants –debug –cdh-cutoff 0.5 –min-domain-number”. We selected a representative species with high genome quality and assembly completeness as the reference genome for each plant lineage. Specifically, we chose *B. napus* of Brassicales, *Citrus medica* of Sapindales, *E. grandis* of Myrtales, *H. brasiliensis* of Malpighiales, *Pyrus bretschneideri* of Rosales, *V. vinifera* of Vitales, *Helianthus annuus* of Asterales, *O. basilicum* of Lamiales, *Capsicum annuum* of Solanales, *S. cereale* of Poales, *Litsea* cubeba of Laurales, *A. trichopoda* of basal_Angiosperms, *T. wallichiana* of gymnosperms, and *S. moellendorffii*, *P. patens*, *M. polymorpha* for non-seed plants.

We performed pairwise synteny region search for BGCs of these representative species and other 69 land plants using the ‘python -m jcvi.compara.synteny mcscan’ and ‘python -m jcvi.formats.base join’ commands with the MCscan (Python version) (Tang et al., 2008) default parameters. Based on the presence of BGCs similar to those in the reference genome on the synteny blocks, we further identified highly conserved BGCs, lineage-specific BGCs, and species-specific BGCs. Microsynteny visualization was prepared using the command ‘python -m jcvi.graphics.synteny’.

## Results

### Distribution and evolution of *TPS* genes in plants

**Figure 1:**
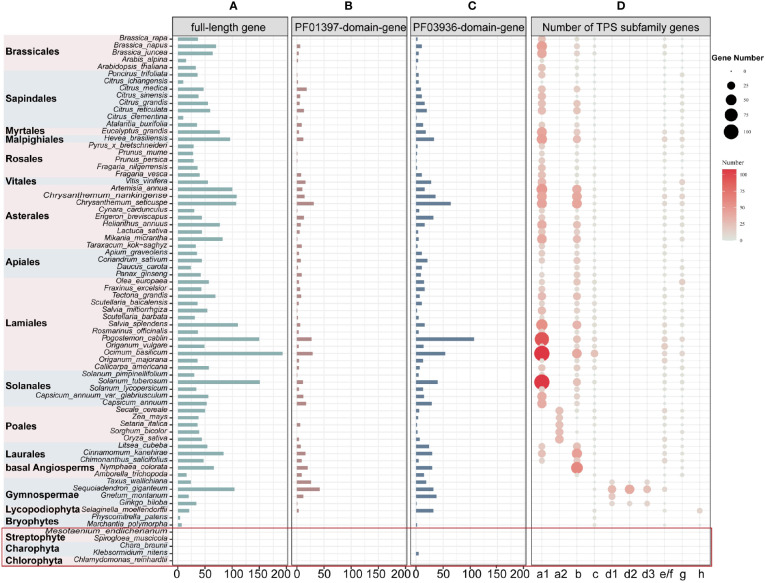
Distribution and classifications of *TPS* genes in 74 studied plant species across 19 orders. **(A-C)** Number of *TPS* genes (including full-length, PF01397-domain-genes, and PF03936-domain-genes). The red square highlights lower plant species. The “full-length gene” represents genes with both “PF01397” and “PF03936” functional domains; “PF01397-domain-gene” and “PF03936-domain-gene” represent plant sequences with only one functional domain of either “PF01397” or “PF03936”. **(D)** The size and color of the bubbles represent the full-length gene number within each subfamily.

After extensive sequences mining, we found that full-length *TPS* genes, PF03936-domain-genes, and PF01397-domain-genes (see Materials and Methods for definition of gene categories) were unevenly distributed among the 74 plants (69 land and 5 lower plant species) (**Supplementary Table 1**). We identified a total of 3,600 full-length *TPS* genes (3,167 of which were longer than 350 amino acids) in all land plant species. Additionally, 513 PF01397-domain-genes were discovered exclusively in seed plant species, and 1,049 PF03936-domain-genes in all land plants except for the moss *Physcomitrella patens* (**Figures 1A–C**; **Supplementary Table 2**). In the five lower plant species, only five PF03936-domain-genes were found in *Klebsormidium nitens* (**Figure 1C**; **Supplementary Table 2**).

To classify the full-length *TPS* genes, we constructed two unrooted *TPS* phylogenetic trees for 3,386 genes from 62 angiosperms and 241 genes from 7 non-angiosperms, respectively (**Supplementary Figures 1**, **2**). Based on the previously characterized genes (**Supplementary Tables 3**, **4**), these genes were classed into the seven subfamilies TPS-a, TPS-b, TPS-c, TPS-d, TPS-e/f, TPS-g, and TPS-h (**Figure 1D**; **Supplementary Figures 1**, **2**; **Supplementary Table 5**). The TPS-c and TPS-e/f subfamilies play a role in primary metabolism and are shared among all angiosperm lineages (**Figure 1D**; **Supplementary Table 6**). However, the divergence of these two subfamilies is inconsistent within both non-seed plants and gymnosperms. The first divergence was observed in liverwort, *Marchantia polymorpha*, while the TPS-e/f subfamily was absent in the moss *P. patens* (**Figure 1D**; **Supplementary Table 6**). Among gymnosperms, *Gnetum montanum* also lacks the TPS-e/f subfamilies (**Figure 1D**; **Supplementary Table 6**). Consistent with previous research (Chen et al., 2011), the TPS-e/f clade in angiosperms underwent a deep divergence, forming two subclades, TPS-f and TPS-e, with TPS-f being dicot-specific (**Supplementary Figure 1**). The divergence of TPS-c and TPS-e/f subfamilies suggests parallel subfunctionalization within each lineage.

The subfamilies TPS-a/b/d/g/h, specialized in secondary metabolism, exhibited lineage-specific differences. The TPS-a/b/g subfamilies were angiosperm-specific, with TPS-a further divided into dicot-specific TPS-a1 and monocot-specific TPS-a2 clades. However, TPS-a was absent in the basal angiosperm species *Nymphaea colorata* and *Amborella trichopoda* (**Figure 1D**; **Supplementary Table 6**), suggesting that TPS-a could represent a novel subfamily that emerged after the divergence of Mesangiospermae from basal angiosperms. TPS-d was gymnosperm-specific and further divided into TPS-d1, TPS-d2, and TPS-d3 clades (**Figure 1D**; **Supplementary Table 6**). The clades TPS-d2 and TPS-d3 were not present in *G. montanum* (**Figure 1D**; **Supplementary Table 6**), potentially indicating a loss of these two sub-clades in *G. montanum* during its evolution or that the subfunctionalization of the TPS-d subfamily after *G. montanum* diverged from other gymnosperms species. TPS-h was identified in both the liverwort *M. polymorpha* and Lycopodiophyta *Selaginella moellendorffii* (**Figure 1D**; **Supplementary Table 6**), suggesting that divergence of the TPS-h clade occurred before the split of liverwort and Lycopodiophyta. In addition, the TPS-b subfamily was absent in monocot species *Oryza sativa* and *Setaria italica*, and the TPS-g subfamily was absent in the dicot species *Apium graveolens* (**Figure 1D**; **Supplementary Table 6**). These absences may have resulted from independent losses of entire the subfamilies in specific plants.

### Prevalent distribution of *TPS* PF03936 and PF01397 domain sequences in microbes

The domain sequences of PF01397 and PF03936 were found to be prevalent among various microbial species (**Supplementary Tables 7**–**9**). Approximately 45% of bacterial families, 26% of archaeal families, and 20% of fungal families were found to possess sequences with PF01397 domain. Meanwhile, around 58% of bacterial families, 48% of archaeal families, and 33% of fungal families contained sequences with PF03936 domain (**Table 1**). Among these sequences, 1.35% to 5.56% of those containing the PF01397 domain and 32.84% to 90.70% of those containing the PF03936 domain were found to be significantly related to conserved terpene synthesis domains with an E-value of 1e-5 (**Table 1**), such as “Isoprenoid_Biosyn_C1 superfamily”, “Terpene_syn_C_2”, “PLN02279 super family (ent-kaur-16-ene synthase)”, “PLN02592 superfamily (ent-copalyl diphosphate synthase)”, “SQHop_cyclase_C superfamily”, “squalene_cyclas superfamily”, and others.

**Table 1:** Statistics of PF03936 or PF01397 domains sequences derived from bacteria, archaea and fungi in the NR database.

			Bacteria	Archaea	Fungi
**NR database**		**Sequences number**	397285288	9525243	27330891
		**Species number**	>115384	>4252	>34345
		**Family number**	>665	>65	>777
**PF03936-domain sequences**	**hmmscan**	**Sequences number**	22789	670	8268
		**Species number**	>7520	>278	>1457
		**Family number**	>383	>31	>256
	**CDD**	**Sequence number**	10894	220	7499
**PF01397-domain sequences**	**hmmscan**	**Sequences number**	7338	234	778
		**Species number**	>2956	>91	>520
		**Family number**	>302	>17	>152
	**CDD**	**Sequence number**	99	13	21
**Full-length genes**	**Gene IDs**	**Family**	**Species**	**CDD domain** **(E-value = 1e-5)**	**Sequence length** **(aa)**
	***WP_145718914.1***	Chitinophagaceae	*Chitinophaga japonensis*	PLN02592 and Terpene_syn_C2	785
	***WP_260645699.1***	Staphylococcaceae	*Staphylococcus aureus*	Terpene_cyclase_plant_C1	584
	***WP_232298052.1***	Nitrosomonadaceae	*Nitrosospira* sp. NpAV	Isoprenoid_Biosyn_C1 superfamily	152
	***KIO47690.1***	Nitrosomonadaceae	*Nitrosospira* sp.	Isoprenoid_Biosyn_C1 superfamily	177

Detailed information of four full-length genes containing both PF01397 and PF03936 domains found in bacteria are shown. “hmmscan” refers to the number of microbial sequences that closely match the Pfam models of PF01397 and PF03936, as identified by hmmscan with E-value = 1e-5; “CDD” indicates the number of sequences of microbes related to terpene synthesis and verified by CDD (https://www.ncbi.nlm.nih.gov/Structure/bwrpsb/bwrpsb.cgi) analysis.

However, full-length *TPS* genes were exceedingly rare in microbes. Only four soil bacterial sequences (*WP_145718914.1*, *WP_260645699.1*, *WP_232298052.1*, *KIO47690.1*) were found to match both the PF03936 and PF01397 domains (**Table 1**). The *WP_145718914.1* and *WP_260645699.1* genes encode potential proteins longer than 500 amino acids (**Table 1**). Further phylogenetic and conservation analysis revealed the *WP_260645699.1* gene shared highly conserved motifs with gymnosperm-specific TPS-d1 genes, while the *WP_145718914.1* gene displayed a region of conserved motifs towards the 5’ end, shared with multiple subfamilies, and demonstrated larger differentiation at the 3’ end (**Supplementary Figure 3A**).

The flanking sequences of the *WP_145718914.1* gene in *Chitinophaga japonensis* shared homologous sequences with 10 *Chitinophaga* species (**Supplementary Figure 3B**; **Supplementary Tables 10**, **11**). After remapping the genomic sequencing data, the gene region of the *WP_145718914.1* showed consistent depth of coverage across the gene body, start and end positions and these gene body coverages were comparable to the genome-wide average (**Supplementary Figure 3C**). This even depth of coverage across the entire gene partially mitigated the impact of genome assembly errors and sequencing contamination. These results supported the presence of *WP_145718914.1*, a plant origin full-length *TPS* genes in a soil bacterium genome, and suggests it may be an intact gene that has been functionally integrated into the genome through horizontal transfer. In contrast, we did not find any homologous fragments of the flanking sequence of the *WP_260645699.1* gene from *Staphylococcus aureus* in the 96 *S. aureus* strains (**Supplementary Table 12**) and 59 *Staphylococcus* genus species (**Supplementary Table 13**). Considering that the sequence in which this gene is located is only 2224 bp long, the presence of the *WP_260645699.1* gene in a bacterial genome remains doubtful.

### Phylogenetic relationships of PF03936 and PF01397 domain sequences

**Figure 2:**
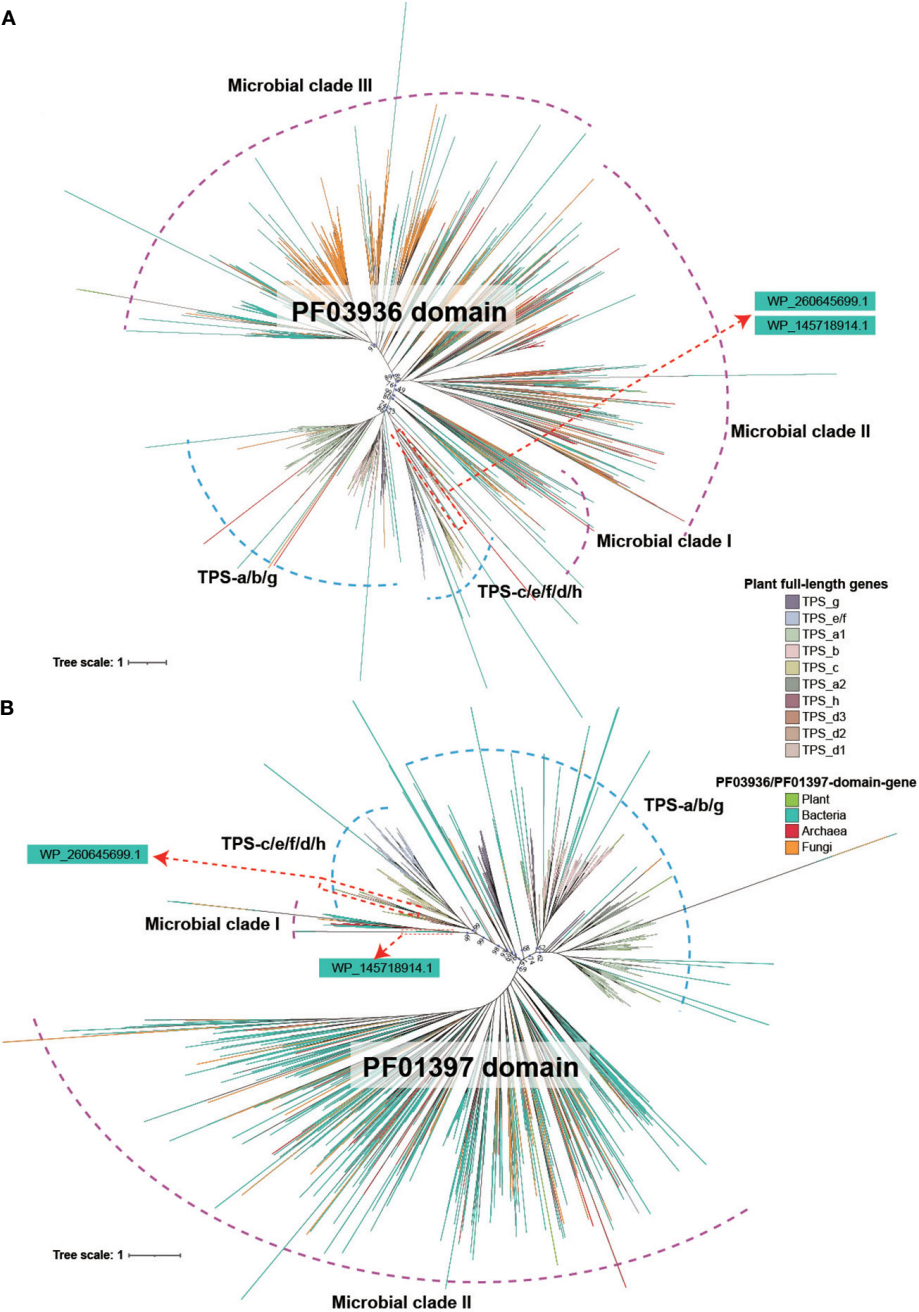
Phylogenetic tree of PF03936 and PF01397 domain sequences in plant and microbial. **(A)** Tree reconstructed based on PF03936 domain sequences from plant, bacteria, archaea and fungi. **(B)** Tree reconstructed based on PF01397 domain sequences from plant, bacteria, archaea and fungi. The color of the branch denotes sequence origins from different biological groups or *TPS* subfamilies.

The phylogenetic relationship of both the PF03936 and PF01397 domain showed a similar topology, indicative of a deep divergence that occurred between plant and microbial (bacteria, archaea and fungi) sequences (**Figures 2A, B**). Deep divergence was also evident among major microbial clades and between angiosperm-specific TPS-a/b/g subfamilies and four other subfamilies (TPS-c, TS-e/f, TPS-d, TPS-h) (**Figures 2A, B**). The phylogenetic relationships of the two *TPS* domain sequences in plants were largely consistent with the full-length *TPS* genes (**Figures 2A, B**; **Supplementary Figures 1**, **2**), suggesting that all full-length *TPS* genes in plants descended from a common ancestor. Additionally, the trees showed that a few plant PF01397/PF03936-domain sequences were located within microbial clades and some microbial sequences were found within plant clades, indicating potential mutual transfer between microbes and plants (**Figures 2A, B**).

Of particular interest is the phylogenetic position of the soil bacterial genes *WP_145718914.1* and *WP_260645699.1*. Both the PF01397 and PF03936 domain sequences of *WP_260645699.1* were located in the gymnosperm-specific TPS-d1 subfamily clade (**Figures 2A, B**). The PF03936 domain of *WP_145718914.1* gene was located in TPS-h clade, while the PF01397 domain was positioned closer to the “Microbial clade I” (**Figures 2A, B**). This further suggests that the *WP_260645699.1* gene is highly conserved with TPS-d subfamily genes. A significant divergence of PF01397 domain was found between *WP_145718914.1* and the plant genes.

### Conserved and lineage-specific divergence of *TPS* genes in land plants

**Figure 3:**
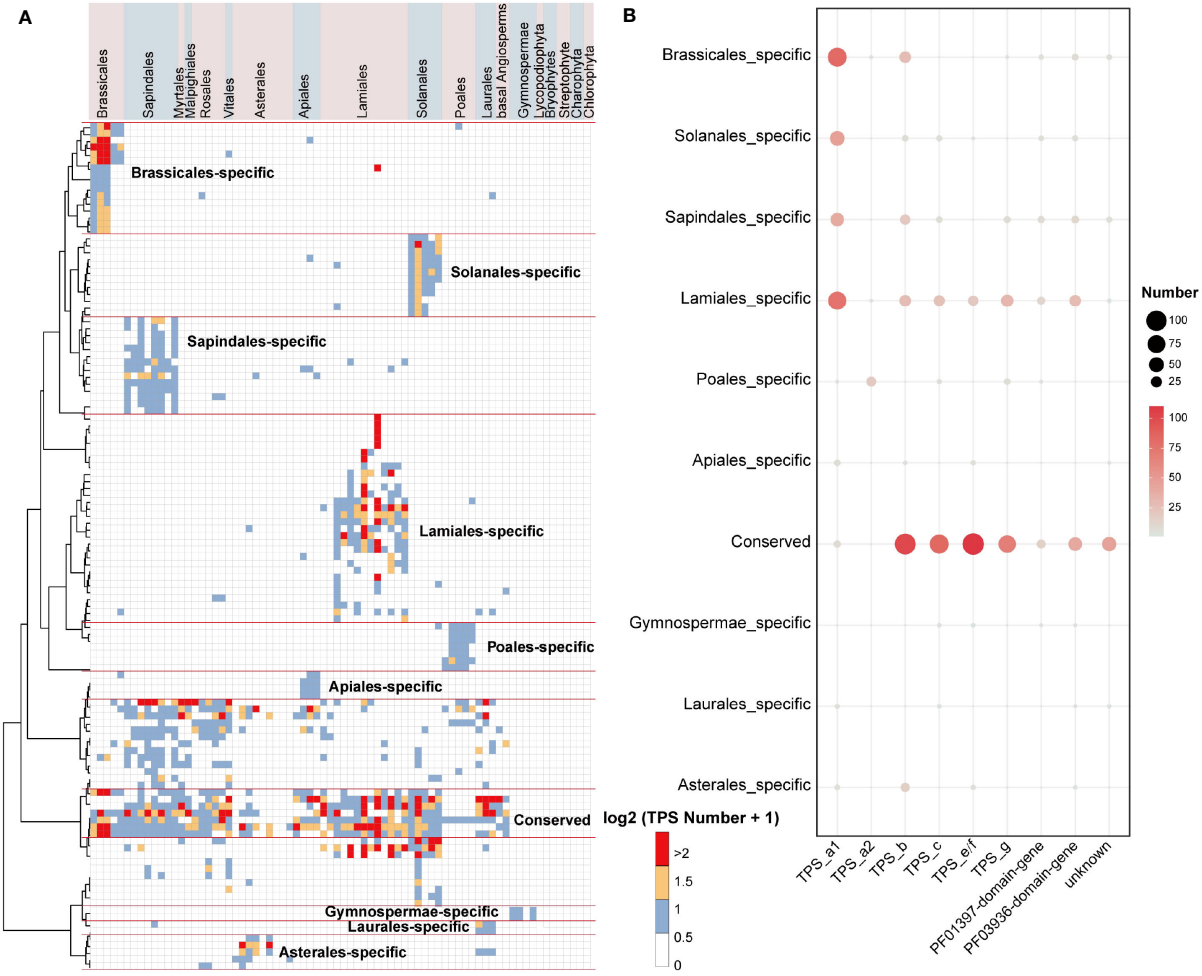
Gene synteny clusters of full-length *TPS* genes in 74 plant species across 19 orders. **(A)** Phylogenetic profiling of the 122 network clusters obtained by infomap (Rosvall and Bergstrom, 2008). The color scale within the heatmap represents the number (log2(gene number + 1)) of full-length *TPS* genes found in each cluster for a given species. Red lines distinguish the specific and conserved *TPS* gene clusters in plant lineages. **(B)** Bubble chart illustrating the number of *TPS* genes in the conserved and lineage-specific clusters. “NA” denotes sequences that have not matched the PF01397 or PF03936 domains. The bubble size and color scheme indicate the gene number.

To shed light on the conserved and lineage-specific evolutionary landscape of *TPS* genes in land plants, we further examined the inter- and intra-specific synteny relationships of plant *TPS* genes. Through gene synteny network clustering, we identified a total of 122 synteny clusters of full-length *TPS* genes, 201 synteny clusters of PF03936-domain-genes, and 121 synteny clusters of PF01397-domain-genes. These clusters revealed a clear conserved and lineage-specific pattern of *TPS* genes among angiosperm plants (**Figure 3A**; **Supplementary Figure 4**).

Only a small number of gymnosperms TPS-c and TPS-e/f genes were found clustered in lineage-specific clusters in the syntenic network, whereas the TPS-h and gymnosperm-specific TPS-d subfamilies were absent from the synteny network (**Figure 3B**; **Supplementary Figure 5**; **Supplementary Table 14**). This is likely due to extreme phylogenetic distance and sampling biases between angiosperms and non-angiosperm species. In the conserved clusters (where cluster genes are shared among multiple lineages), the genes mainly belong to the TPS-c, TPS-e/f, TPS-b, and TPS-g subfamilies. Additionally, the Lamiales, Solanales, Sapindales, and Brassicales lineage-specific clusters (where cluster genes are primarily shared in only one lineage) mainly consist of TPS-a1 genes, and the Poales lineage-specific clusters primarily contain TPS-a2 genes, and the Asterales lineage-specific clusters mostly consist of TPS-b genes (**Figure 3B**; **Supplementary Table 14**). In the Lamiales, which include important spice plants, we found that the lineage-specific cluster contains a large number of TPS-a subfamily genes along with many TPS-b, TPS-c, TPS-e/f, and TPS-g subfamilies genes (**Figures 3A, B**). This may be a key indication of the diversity of terpenes in this lineage.

**Figure 4:**
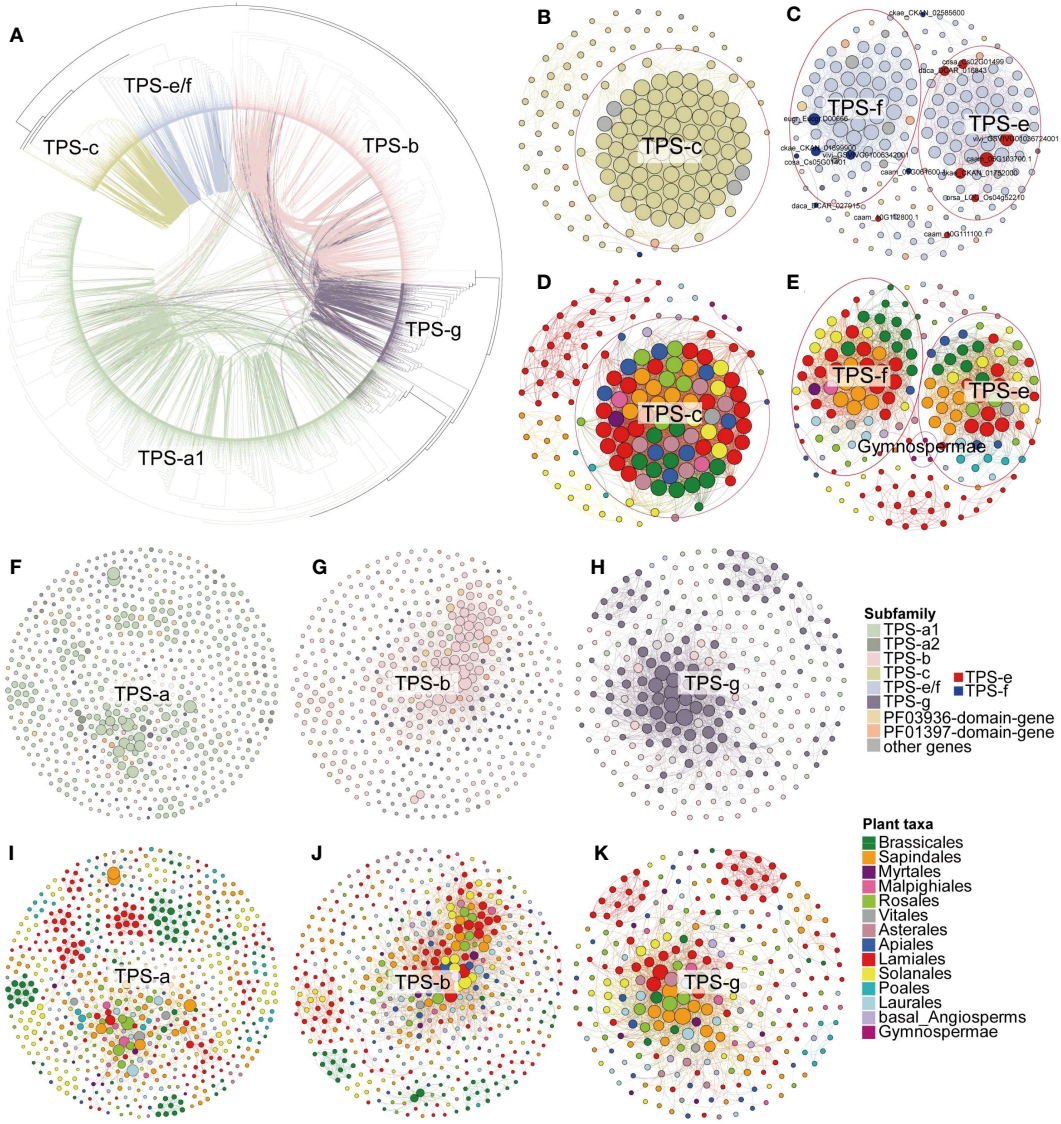
Syntenic relationships of full-length *TPS* genes. **(A)** Maximum-likelihood tree of full-length *TPS* genes from angiosperms, depicting their syntenic relationships. Each connecting line inside the circular gene tree represents a syntenic relationship between two genes, with lines color-coded for specific *TPS* subfamilies. **(B, C, F–H)** Synteny subnetworks of TPS-c, TPS-e/f, TPS-a, TPS-b, and TPS-g subfamily, respectively, with node colors (color panel) representing the different gene subfamily assignments. The red and blue nodes in **(C)** indicate TPS-e and TPS-f subfamily genes with established gene classifications (**Supplementary Table 4**). **(D, E, I–K)** Synteny subnetworks of the TPS-c, TPS-e/f, TPS-a, TPS-b, and TPS-g subfamily, respectively, with node colors denoting the different plant lineages (color panel for species order). Genes circled in red for panels **(B–E)** mark the inter-lineage conserved TPS-c, TPS-e, and TPS-f synteny clusters.

These conserved and lineage-specific clusters were clearly evident in each subfamily subnetwork (**Figures 4B–K**; **Supplementary Figure 5**), demonstrating that *TPS* genes have undergone duplication and functional divergence in different plant lineages over evolutionary time. In the TPS-c subfamilies subnetwork, we identified one major cluster contains genes from all the angiosperm lineages, indicating broad conservation across flowering plants. We also found five lineage-specific clusters composed of genes belonging only to Lamiales, Solanales, Sapindales, Laurales, and Gymnospermae, respectively (**Figures 4B, D**). The TPS-f subfamily is derived from the TPS-e subfamily and has been identified as TPS-e/f (Chen et al., 2011). In our TPS-e/f subnetwork, the previously characterized TPS-e and TPS-f related genes (for gene details, see **Supplementary Table 4**) were found in two distinct clusters without any synteny relationships between them. This suggests functional divergence of the duplicated genes (**Figures 4C, E**). Notably, the TPS-e cluster genes were found in all angiosperm lineages, while monocots lacked genes in the TPS-f cluster (**Figures 4C, E**). This further indicates that TPS-f subfamilies may have evolved independently in dicots after the divergence of monocots and dicots.

We further examined pairwise syntenic relationships among the full-length *TPS* genes in angiosperms on the phylogenetic tree (Letunic and Bork, 2007). Compared to the TPS-c and TPS-e/f subfamilies, we detected more synteny relationships among genes of angiosperm-specific TPS (a/b/g) subfamilies (**Figure 4A**). Although TPS-c and TPS-e/f were closely related in phylogeny, links between genes of these two subfamilies were rare (**Figure 4A**). Interestingly, in some cases, connections were found between genes from the distal gene clades TPS-e/f and TPS-a/b/g (**Figure 4A**). One possible interpretation is that TPS-c and TPS-e/f diverged in early land plants, while the most common ancestral gene of TPS-a, TPS-b, and TPS-g expanded after the split of the gymnosperm and angiosperm lineages, resulting in more gene synteny between them. Moreover, 93 PF03936-domain-genes and 42 PF01397-domain-genes shared the synteny relationship with full-length genes (**Figure 3B**; **Supplementary Figure 5**; **Supplementary Table 14**), which implies that these genes might have originated from the domain loss of the full-length genes.

### The evolutionary footprint of terpene-related BGCs

We performed a comprehensive inter-specific BGC homology analysis among 74 plants (**Supplementary Table 1**) to investigate the evolutionary footprint of terpene-related BGCs. We identified a total of 512 terpene-related BGCs, including 380 terpene BGCs, 8 sesterterpene BGCs, and 124 hybrid BGCs (**Supplementary Tables 15**–**17**). Among these BGCs, 333 (65.0%) contained full-length *TPS* genes, 97 (9.2%) contained PF03936/PF01397-domain-genes, and 132 (25.8%) were without *TPS* related genes (**Supplementary Figure 6**; **Supplementary Table 18**). Through comparative genomic analysis, we have discovered that these *TPS* containing BGCs are either highly conserved across multiple lineages (highly conserved BGCs), conserved within a single lineage (lineage-specific BGCs), or specific to individual species (species-specific BGCs) (**Supplementary Tables 19**–**21**). These BGCs distinctly display the footprints of their formation and diversification during plant evolution.

**Figure 5:**
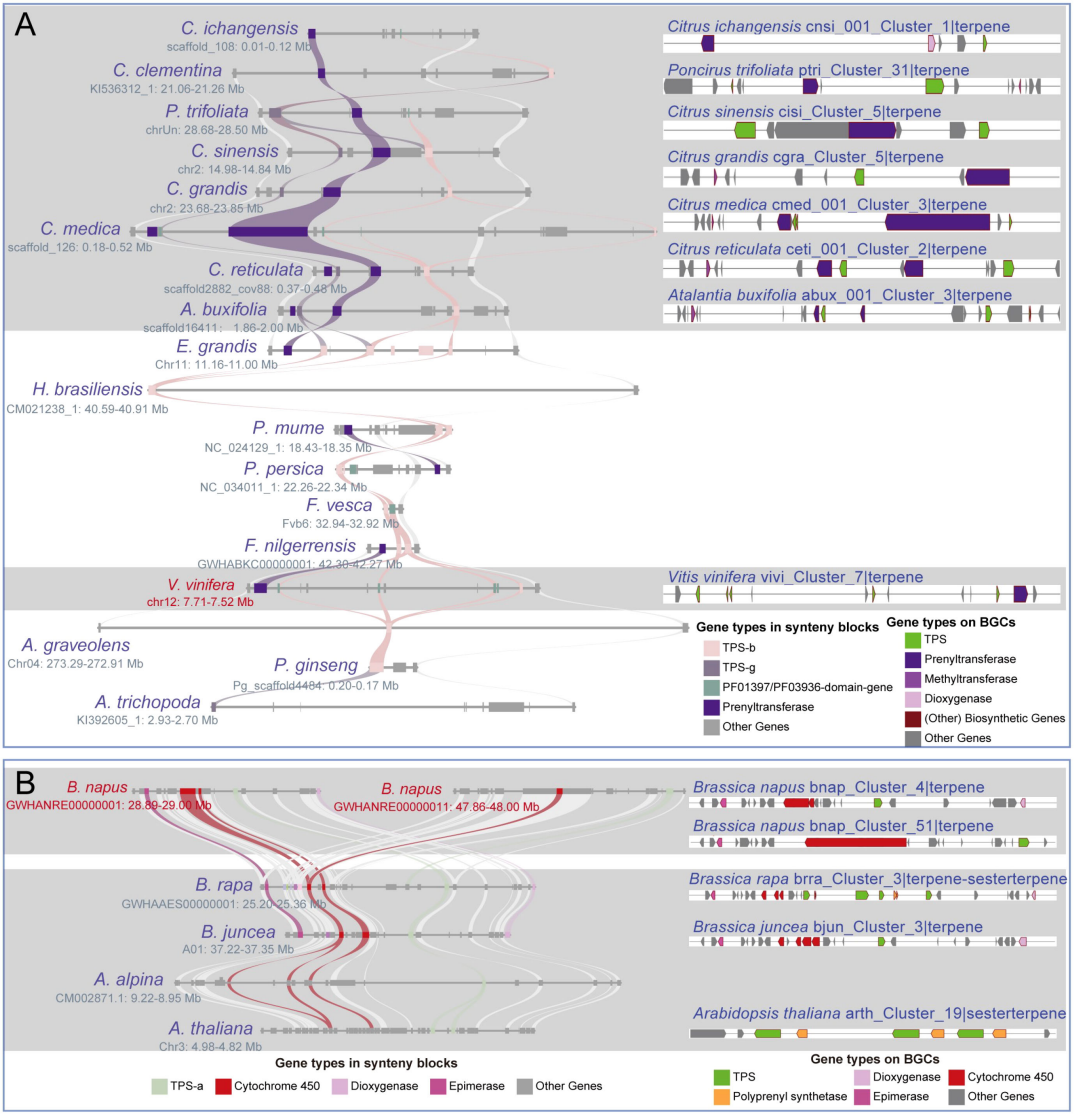
Differentiation of the homologous BGCs. **(A)** The “vivi_Cluster_7|terpene” BGC in *V. vinifera* shares synteny blocks with 17 angiosperms from 6 lineages, and 7 homologous BGCs are identified within seven Sapindales species. **(B)** The “bnap_Cluster_4|terpene” and “bnap_Cluster_4|terpene” BGCs share synteny blocks with 4 Brassicales species, and 3 homologous BGCs are identified within these synteny blocks. Both **(A, B)** demonstrate homologous BGCs within varying differentiation.

**Figure 6:**
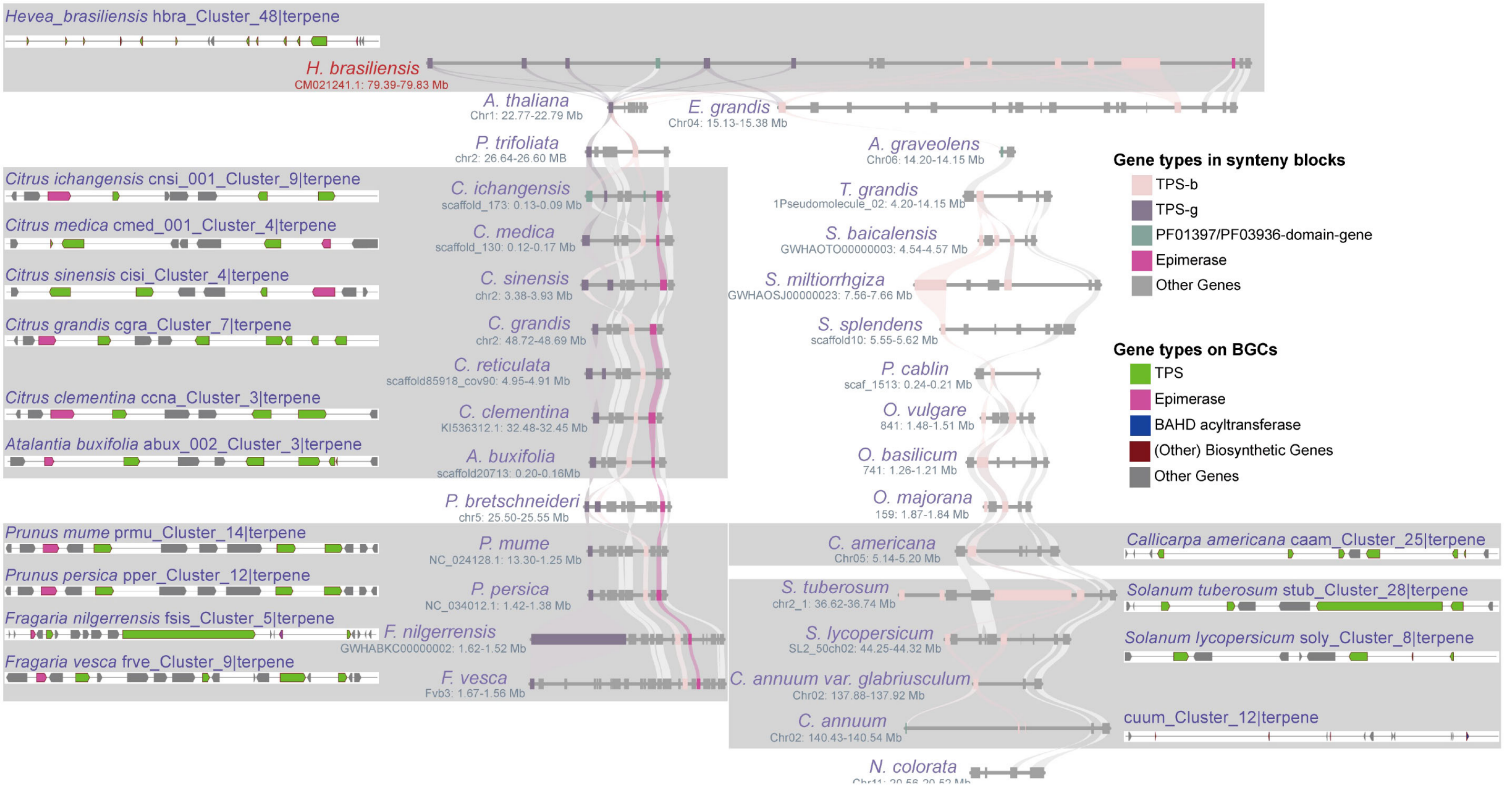
Differentiation of homologous BGCs. The “hbra_Cluster_48|terpene” BGC in *H. brasiliensis* shares synteny blocks with 31 angiosperms from 19 lineages, and its 15 homologous BGCs were identified within the synteny blocks of Sapindales, Rosales, Lamiales, and Solanales species, demonstrating the differentiation of homologous BGCs in terms of both the number and type of metabolic genes.

In this study, we chose three highly conserved BGCs and investigated their evolutionary differentiation (**Supplementary Table 19**). The “vivi_Cluster_7|terpene” BGC was found in *Vitis vinifera* and shares a conserved syntenic block with 17 angiosperm species. Moreover, seven homologous BGCs were found only in the syntenic blocks of Sapindales species (**Figure 5A**; **Supplementary Table 19**). The numbers and sequences of *TPS* and *prenyltransferase (PT)* genes in these homologous BGCs show remarkable divergence (**Figure 5A**; **Supplementary Figure 7**; **Supplementary Table 19**). Both *TPS* and *PT* are involved in the terpene synthesis pathway. The “hbra_Cluster_48|terpene” BGC in *Hevea brasiliensis* shares conserved syntenic blocks with 30 angiosperm species, and 14 conserved homologous BGCs were identified in the syntenic blocks of six Sapindales, four Rosales, one Lamiales, and three Solanales species (**Figure 6**; **Supplementary Table 19**). All homologous BGCs from Lamiales and Solanales species lost the TPS-g and Epimerase genes (**Figure 6**; **Supplementary Table 19**). The “eugr_Cluster_22|terpene” BGC in *Eucalyptus grandis* has a conserved syntenic block with 55 angiosperms and one gymnosperm, and 10 homologous BGCs were identified in four Sapindales, one Malpighiales, and five Rosales species (**Supplementary Table 19**). In these homologous BGCs, significant TPS-a1 gene tandem duplication and loss of metabolic genes were also observed (**Supplementary Table 19**).

The unique full-length gene *vivi_GSVIVG01000401001* (*VvTPS34*) within the conserved “vivi_Cluster_7|terpene” terpene BGC in *V. vinifera* was functionally characterized as an (*E*)-β-Ocimene synthase (Martin et al., 2010). (*E*)-β-Ocimene is a commonly observed monoterpene (Fäldt et al., 2003), suggesting this conserved BGC and its homologous BGCs may be involved in the biosynthesis of this monoterpene. Further potential functional prediction of the full-length *TPS* genes in the “eugr_Cluster_22|terpene” BGC aligned to (+)-delta-cadinene synthase in UniprotKB (https://www.uniprot.org/) with 72-100% identity (**Supplementary Table 20**), indicating this BGC and homologous BGCs may be involved in the biosynthesis of this sesquiterpene. The genes within the conserved “hbra_Cluster_48|terpene” BGC showed no high sequence similarity to any characterized proteins in UniprotKB, precluding functional prediction. Thus, the potential function of this conserved BGC remains unknown.

We observed variations in the number of metabolic genes associated with the divergence of homologous BGCs in the single lineage (**Supplementary Table 21**). For instance, both the “bnap_Cluster_51|terpene” and “bnap_Cluster_4|terpene” BGCs of *Brassica napus* are sharing synteny blocks with three homologous BGCs of *B. rapa*, *B. juncea* and *A. thaliana* (**Figure 5B**). Among these homologous BGCs, the number of cytochrome 450 genes is inconsistent, while the polyprenyl synthetase, epimerase, and dioxygenase genes are lost in some BGCs (**Figure 5B**). Insertion of new metabolic genes and frequent tandem duplications of *TPS* genes, especially the lineage-specific expansion of angiosperms-specific TPS-a/b/g subfamilies, may be associated with the formation of these species-specific BGCs. Examples of these BGCs include the tandem duplication of TPS-d3 and/or Cytochrome 450 genes in “tawa_Cluster_14|terpen” and “tawa_Cluster_48|terpen BGCs”, the TPS-f genes expansion in *Secale cereale*, and TPS-a1 genes expansion in *Ocimum basilicum* (**Supplementary Figure 8**; **Supplementary Table 22**).

## Discussion

### HGT and gene fusion origin of *TPS* genes

HGT of the microbial genes to plants is believed to occur frequently, and has facilitated the transition from aquatic to terrestrial environments for land plants (Ma et al., 2022; Zhang et al., 2022). Gene fusion is an important driver in the evolution of multidomain proteins (Man et al., 2020). It has been reported that HGT and gene fusion may collectively contribute to the origination of full-length *TPS* gene in land plants. Based on extensive microbial sampling and sequence survey, we found that many PF01397 and PF03936 domain sequences shared similarity across different kingdoms, confirming an early origin of these two domains and the potential for multiple instances of gene transfer among microbes and plants.

However, full-length *TPS* gene were very rare or absent in microbes and lower plants. Thus, we concluded that the fusion of PF03936 (C-terminal) and PF01397 (N-terminal) domains more likely occurred in the ancestral land plant after the divergence from lower plants, and these two domains could have been individually acquired from microbe via HGT. Our sequence analysis found one case that a potential full-length *TPS* gene may derive by HGT from plants to soil bacteria, suggesting the HGT of *TPS* is possibly also a pathway for terpene innovation in microbes.

### Conservation and differentiation dynamics of *TPS* subfamilies

Terpenes involved in primary metabolism are synthesized through relatively conserved pathways in plants (Yang et al., 2020). Our results reveal that the TPS-c and TPS-e/f subfamilies, which are involve in GA production, display high gene synteny conservation among angiosperms with small-scale lineage-specific expansions. In contrast, the angiosperm-specific TPS-a/b/g subfamilies, dedicated to secondary metabolism, exhibit significant lineage-specific patterns. *TPS* genes possess profound functional plasticity, where minor changes in active sites can dramatically affect catalytic properties, allowing for the emergence of new functions with minimal investment in evolving new enzymes (Karunanithi and Zerbe, 2019). The evolution of the ability to synthesize specialized metabolites has likely been crucial for the survival and diversification of various plant species (Qi et al., 2004). Consequently, the expansion and subfunctionalization/neofunctionalization of lineage-specific *TPS* genes facilitate the production of various terpene metabolites in response to changing biotic and abiotic environments, contributing to the differentiation of plant ecotypes (Tholl, 2015; Pichersky and Raguso, 2018; Jiang et al., 2019). The diversity of PVTs also leads to significantly variable compositions of oils produced in various plants.

Angiosperms have undoubtedly dominated recent ecological history on land by frequent gene duplication (Zhang et al., 2022), polyploidy events, chromosome reorganization, and other molecular mechanisms that create a rich source of genetic novelty and adaptation to complex environments. These activities around genome structural variations may have contributed to the significant expansion and lineage-specific diversification of TPS-a/b/g subfamilies in angiosperms. Phylogenetic studies also show the deep divergence between angiosperm-specific subfamily TPS-a/b/g and other four subfamilies (TPS-c, TPS-e/f, TPS-d, TPS-h). Thus, the subfunctionalization/neofunctionalization of TPS-a/b/g subfamilies in various angiosperms lineages led to a diversity of enzymes, contributing to significant terpene and species phenotypic diversity.

Gain and loss of protein domains are widespread evolutionary events (Man et al., 2020) that can generate novel genetic diversity and further species diversity. By revealing the relationships of the PF03936/PF01397-domain-genes and full-length genes in plants, this study sheds light on the complex evolutionary trajectories of plant *TPSs* and how this domain gain and loss has been involved in shaping the terpene chemical diversity. However, it should be noted these findings are susceptible to gene annotation errors, which must be carefully considered in future studies. The broad distribution of PF03936/PF01397-domain-genes across all *TPS* subfamily clades, along with their syntenic relationships with full-length *TPS* genes, supports the hypothesis that these two domains can be lost in some full-length *TPS* genes. In addition to domain shuffling and loss, the outright loss of entire subfamilies during genome evolution of individual species represents another mechanism contributing to the inter-specific diversity of terpene profiles.

### Terpene-related BGCs

The establishment and maintenance of BGCs has been driven by natural selection, including long-term purifying selection, positive selection, and balancing selection (Wu et al., 2022). Thus, the conserved BGCs imply a stronger selective advantage in the genome (Kautsar et al., 2017). In this study, we identified three conserved terpene BGCs as highly conserved synteny blocks that are shared among multiple plant lineages, suggesting that secondary metabolic terpenes produced by these BGCs may play a crucial role in defense or interactions with the external environment of these plant lineages. There is compelling evidence that BGCs in plants arose from gene duplication, neofunctionalization, genomic relocation, and chromosomal inversion (Nützmann et al., 2018; Rokas et al., 2018; Liu et al., 2020; Wu et al., 2022). We found that gene tandem duplication, loss, or gain of metabolic genes were associated with the divergence of homologous BGCs. Moreover, lineage-specific expansion of *TPS* genes and the insertion of new metabolic genes are probably the primary pathways for the formation of species-specific BGCs. These homologous or specific BGCs enrich the diversity of plant terpene metabolites and facilitate plants’ survival advantage in different ecological niches. However, further investigation into the functions of these BGCs are needed to better understand the relationship between BGCs and plant terpene metabolites.

This study employed comprehensive phylogenetic samplings and analysis of terpene synthase sequences from plants and microbes to elucidate the origin and diversification of *TPS* genes in land plants. We revealed gene fusion of PF01397 and PF03936 domain occurred in early land plants, and the synteny relationships and diversification of *TPS* genes across major land plant groups. Additionally, we identified plant *TPS* gene synteny relationships and the evolution of homologous and species-specific terpene-relate BGCs. Our study thus provides new and insightful perspectives into the diversity of terpene biosynthesis in plants and presents invaluable resources for future evolutionary and functional studies. This could also enhance our understanding and further inform the biotechnology industry on how to optimize the utilization of oil-producing plants.

### Statistics and reproducibility

No statistical tests were employed in this study. All methods used for sequence analyses are described in the corresponding methods.

## Data availability statement

Publicly available datasets were analyzed in this study. This data can be found here: All single domain and full-length *TPS* sequences obtained from various microbes and plants and the sequence alignments (PF01397 domain, PF03936 domain and full-length gene sequences) could be accessed on GitHub at the following link: https://github.com/xmy-1682/TPS-data/tree/master/sequences_data and https://github.com/xmy-1682/TPS-data/tree/master/Sequence_alignments. The Newick In review format of all phylogenetic trees (in **Figures 2**, **3**, **Supplementary Figures 1**, **2**) generated in this study are available on GitHub at: https://github.com/xmy-1682/TPSdata/tree/master/Newick_format_of_phylogenetic_trees. The raw data used for regenerating the gene synteny network can be found at: https://github.com/xmy 1682/TPS-data/tree/master/Raw_data. All terpene-related BGCs identified in 72 plant species are listed in Supplementary Table 15, and could be accessed on GitHub at: https://github.com/xmy-1682/TPS-data/tree/master/PlantiSMASH_results].

## Author contributions

J-FM: Methodology, Supervision, Writing – review & editing, Writing – original draft. X-MY: Data curation, Formal Analysis, Investigation, Methodology, Project administration, Validation, Visualization, Writing – original draft, Writing – review & editing. S-SZ: Investigation, Visualization, Writing – review & editing. HL: Investigation, Visualization, Writing – review & editing. S-WZ: Visualization, Writing – review & editing. X-CT: Visualization, Writing – review & editing. T-LS: Visualization, Writing – review & editing. Y-TB: Visualization, Writing – review & editing. Z-CL: Visualization, Writing – review & editing. K-HJ: Visualization, Writing – review & editing. SN: Visualization, Writing – review & editing. J-FG: Visualization, Writing – review & editing. LK: Visualization, Writing – review & editing. IP: Investigation, Supervision, Writing – review & editing.
