# Supplementary material for: Unraveling the evolutionary dynamics of the TPS gene family in land plants
Source: Front Plant Sci. 2023 Oct 12;14:1273648. doi: 10.3389/fpls.2023.1273648 (PMC10600500; doi:10.3389/fpls.2023.1273648)
Supplement: Supplementary file 1 [file DataSheet_1.zip › Supplementary Figures.doc]

**Supplementary Fig. 1.** **Phylogenetic tree of full-length *TPS* genes from 62 angiosperms belonging to 13 orders.** Including 5 Brassicales species, 8 Sapindales species, 1 Myrtales species, 1 Malpighiales species, 5 Rosales species, 1 Vitales species, 9 Asterales species, 4 Apiales species, 13 Lamiales species, 5 Solanales species, 5 Poales species, 3 Laurales species, and 2 basal Angiosperm species.

 **Supplementary Fig. 2.** **Phylogenetic tree of full-length *TPS* genes from** **gymnosperms (*T. wallichiana*, *S. giganteum*, *G. montanum*, *G. biloba*), non-seed plants (*S. moellendorffii*, *P. patens*, *M. polymorpha*), *A. thaliana* and bacteria.** The gymnosperm-specific TPS-d subfamily was divided into the TPS-d1, TPS-d2, and TPS-d3 clades, referencing genes reported by Martin *et al*., 2010 and *A. thaliana* *TPS* genes ^1,2^ for classification of TPS-a, TPS-b, TPS-c, TPS-e/f and TPS-g subfamilies (Supplementary Table 3 and Supplementary Table 4).

**Supplementary Fig. 3. The phylogenetic relationship and homologous sequence analysis of full-length genes *WP_145718914.1* and *WP_260645699.1* in soil bacteria.** (A) Phylogenetic relationship and conserved motifs of the *WP_145718914.1* and *WP_260645699.1*, compared with plant full-length genes. (B) Homologous sequences of the upstream and downstream of gene *WP_145718914.1,* blasted against the genomes of 10 species from the *Chitinophaga* genus. (C) Following the mapping of raw sequencing data to the *C. japonensis* genome, the read coverage around the *WP_145718914.1* is visualized.

**Supplementary Fig. 4. Phylogenetic profiling of gene synteny sharing in PF01397-domain-genes and PF03936-domain-genes for 74 plant species across 19 orders.** Phylogenetic profiling of the 201 network clusters of PF03936-domain-genes and 121 network clusters of PF01397-domain-genes were obtained using infomap ^3^. The color scale in the heatmap represents the number (log2(gene number + 1)) of PF03936/PF01397-domain-genes found in each cluster for a given species.

**Supplementary Fig. 5.** **Gene synteny network for the full-length *TPS* gene family based on all detected syntenic relationships in 74 plant species across 19 orders.** Communities (subnetworks) identification are based on the clique percolation method (*k* = 3). The size of each node (bubble) indicates the number of connections (edges) a specific gene has. Subnetworks are labeled according to their subfamily membership.

**Supplementary Fig. 6. Number of biosynthetic gene clusters (BGCs) for the three different *TPS* gene categories identified in the 74 plant species spanning 19 orders sampled.** The different colors signify the BGCs with the different *TPS* gene categories (full-length *TPS* genes, PF01397/PF03936-domain-genes, absence *TPS* genes).

 **Supplementary Fig. 7. Conserved domains identified of *PT* genes.** The conserved domains were identified by CDD (<https://www.ncbi.nlm.nih.gov/Structure/bwrpsb/bwrpsb.cgi>) for *PT* genes in “vivi_Cluster_7|terpene” and their homologous BGCs.

**Supplementary Fig. 8. Synteny blocks of species-specific BGCs.** (A-B) The *T. wallichiana*-specific BGCs “tawa_Cluster_14|terpen” and “tawa_Cluster_48|terpen” share synteny blocks with *S. giganteum* and *G. biloba*. (C) The *B. napus*-specific BGC “bnap_Cluster_14|terpene” shares synteny with *B. rapa* and *B. juncea*. (D) The *S. cereale*-specific BGC of “scer_Cluster_12|terpene” shares synteny blocks with *Z. mays*, *S. italica, S. bicolor and* *O. sativa*. Compared to the synteny blocks, these species-specific BGCs demonstrate gene tandem duplication, translocation, or loss of metabolic genes.

**References**
